# Supplementary material for: Quality of Life and Patient-Reported Outcomes Following Proton Therapy for Oropharyngeal Carcinoma: A Systematic Review
Source: Cancers (Basel). 2023 Apr 12;15(8):2252. doi: 10.3390/cancers15082252 (PMC10137022; doi:10.3390/cancers15082252)
Supplement: Supplementary file 1 [file cancers-15-02252-s001.zip › cancers-2117521-supplementary.pdf]

Supplementary A: PRISMA Checklist

Section and Topic	Item #	Checklist item	Location where item is reported
**TITLE**			
Title	1	Identify the report as a systematic review.	Title
**ABSTRACT**			
Abstract	2	See the PRISMA 2020 for Abstracts checklist.	Attached
**INTRODUCTION**			
Rationale	3	Describe the rationale for the review in the context of existing knowledge.	Intro, Para 4
Objectives	4	Provide an explicit statement of the objective(s) or question(s) the review addresses.	Intro, Para 4
**METHODS**			
Eligibility criteria	5	Specify the inclusion and exclusion criteria for the review and how studies were grouped for the syntheses.	MATERIALS AND METHODS; Systematic review protocol and eligibility criteria
Information sources	6	Specify all databases, registers, websites, organisations, reference lists and other sources searched or consulted to identify studies. Specify the date when each source was last searched or consulted.	MATERIALS AND METHODS; Search strategy and selection process
Search strategy	7	Present the full search strategies for all databases, registers and websites, including any filters and limits used.	Supplementary Materials
Selection process	8	Specify the methods used to decide whether a study met the inclusion criteria of the review, including how many reviewers screened each record and each report retrieved, whether they worked independently, and if applicable, details of automation tools used in the process.	MATERIALS AND METHODS; Search strategy and selection process
Data collection process	9	Specify the methods used to collect data from reports, including how many reviewers collected data from each report, whether they worked independently, any processes for obtaining or confirming data from study investigators, and if applicable, details of automation tools used in the process.	MATERIALS AND METHODS; Search strategy and selection process
Data items	10a	List and define all outcomes for which data were sought. Specify whether all results that were compatible with each outcome domain in each study were sought (e.g. for all measures, time points, analyses), and if not, the methods used to decide which results to collect.	MATERIALS AND METHODS; Search strategy and selection process<br>Supplementary Materials
	10b	List and define all other variables for which data were sought (e.g. participant and intervention	MATERIALS AND

Section and Topic	Item #	Checklist item	Location where item is reported
		characteristics, funding sources). Describe any assumptions made about any missing or unclear information.	METHODS; Data review, extraction and synthesis
Study risk of bias assessment	11	Specify the methods used to assess risk of bias in the included studies, including details of the tool(s) used, how many reviewers assessed each study and whether they worked independently, and if applicable, details of automation tools used in the process.	MATERIALS AND METHODS; Quality assessment
Effect measures	12	Specify for each outcome the effect measure(s) (e.g. risk ratio, mean difference) used in the synthesis or presentation of results.	Table 2
Synthesis methods	13a	Describe the processes used to decide which studies were eligible for each synthesis (e.g. tabulating the study intervention characteristics and comparing against the planned groups for each synthesis (item #5)).	MATERIALS AND METHODS; Meta-analyses
	13b	Describe any methods required to prepare the data for presentation or synthesis, such as handling of missing summary statistics, or data conversions.	NR
	13c	Describe any methods used to tabulate or visually display results of individual studies and syntheses.	MATERIALS AND METHODS; Data review, extraction and synthesis
	13d	Describe any methods used to synthesize results and provide a rationale for the choice(s). If meta-analysis was performed, describe the model(s), method(s) to identify the presence and extent of statistical heterogeneity, and software package(s) used.	MATERIALS AND METHODS; Data review, extraction and synthesis
	13e	Describe any methods used to explore possible causes of heterogeneity among study results (e.g. subgroup analysis, meta-regression).	NR
	13f	Describe any sensitivity analyses conducted to assess robustness of the synthesized results.	NR
Reporting bias assessment	14	Describe any methods used to assess risk of bias due to missing results in a synthesis (arising from reporting biases).	NR
Certainty assessment	15	Describe any methods used to assess certainty (or confidence) in the body of evidence for an outcome.	NR
**RESULTS**			
Study selection	16a	Describe the results of the search and selection process, from the number of records identified in the search to the number of studies included in the review, ideally using a flow diagram.	Fig 1, RESULTS; Study selection and quality assessment
	16b	Cite studies that might appear to meet the inclusion criteria, but which were excluded, and explain why they were excluded.	NR
Study characteristics	17	Cite each included study and present its characteristics.	Table 1

Section and Topic	Item #	Checklist item	Location where item is reported
Risk of bias in studies	18	Present assessments of risk of bias for each included study.	RESULTS; Study selection and quality assessment, Supplement
Results of individual studies	19	For all outcomes, present, for each study: (a) summary statistics for each group (where appropriate) and (b) an effect estimate and its precision (e.g. confidence/credible interval), ideally using structured tables or plots.	Table 3
Results of syntheses	20a	For each synthesis, briefly summarise the characteristics and risk of bias among contributing studies.	RESULTS; Study selection and quality assessment,
	20b	Present results of all statistical syntheses conducted. If meta-analysis was done, present for each the summary estimate and its precision (e.g. confidence/credible interval) and measures of statistical heterogeneity. If comparing groups, describe the direction of the effect.	RESULTS; Studies comparing proton therapy and photon radiotherapy<br>RESULTS: Effect of time<br>RESULTS: Effect of dose factors
	20c	Present results of all investigations of possible causes of heterogeneity among study results.	RESULTS: QOL and PRO measurements
	20d	Present results of all sensitivity analyses conducted to assess the robustness of the synthesized results.	NR
Reporting biases	21	Present assessments of risk of bias due to missing results (arising from reporting biases) for each synthesis assessed.	NR
Certainty of evidence	22	Present assessments of certainty (or confidence) in the body of evidence for each outcome assessed.	NR
**DISCUSSION**			
Discussion	23a	Provide a general interpretation of the results in the context of other evidence.	Discussion, Para 1
	23b	Discuss any limitations of the evidence included in the review.	Discussion, Para 5
	23c	Discuss any limitations of the review processes used.	Discussion, Para 5
	23d	Discuss implications of the results for practice, policy, and future research.	Conclusion
**OTHER INFORMATION**			
Registration and protocol	24a	Provide registration information for the review, including register name and registration number, or state that the review was not registered.	MATERIALS AND METHODS; Systematic review protocol and eligibility criteria

Section and Topic	Item #	Checklist item	Location where item is reported
	24b	Indicate where the review protocol can be accessed, or state that a protocol was not prepared.	MATERIALS AND METHODS; Systematic review protocol and eligibility criteria
	24c	Describe and explain any amendments to information provided at registration or in the protocol.	NR
Support	25	Describe sources of financial or non-financial support for the review, and the role of the funders or sponsors in the review.	Compliance with Ethical Standards
Competing interests	26	Declare any competing interests of review authors.	Compliance with Ethical Standards
Availability of data, code and other materials	27	Report which of the following are publicly available and where they can be found: template data collection forms; data extracted from included studies; data used for all analyses; analytic code; any other materials used in the review.	NR

From: Page MJ, McKenzie JE, Bossuyt PM, Boutron I, Hoffmann TC, Mulrow CD, et al. The PRISMA 2020 statement: an updated guideline for reporting systematic reviews. BMJ 2021;372:n71. doi: 10.1136/bmj.n71

For more information, visit: http://www.prisma-statement.org/

Section and Topic	Item #	Checklist item	Reported (Yes/No)
**TITLE**			
Title	1	Identify the report as a systematic review.	Yes
**BACKGROUND**			
Objectives	2	Provide an explicit statement of the main objective(s) or question(s) the review addresses.	Yes
**METHODS**			
Eligibility criteria	3	Specify the inclusion and exclusion criteria for the review.	
Information sources	4	Specify the information sources (e.g. databases, registers) used to identify studies and the date when each was last searched.	Yes
Risk of bias	5	Specify the methods used to assess risk of bias in the included studies.	
Synthesis of results	6	Specify the methods used to present and synthesise results.	Yes

Section and Topic	Item #	Checklist item	Reported (Yes/No)
**RESULTS**			
Included studies	7	Give the total number of included studies and participants and summarise relevant characteristics of studies.	Yes
Synthesis of results	8	Present results for main outcomes, preferably indicating the number of included studies and participants for each. If meta-analysis was done, report the summary estimate and confidence/credible interval. If comparing groups, indicate the direction of the effect (i.e. which group is favoured).	Yes
**DISCUSSION**			
Limitations of evidence	9	Provide a brief summary of the limitations of the evidence included in the review (e.g. study risk of bias, inconsistency and imprecision).	Yes
Interpretation	10	Provide a general interpretation of the results and important implications.	Yes
**OTHER**			
Funding	11	Specify the primary source of funding for the review.	Yes
Registration	12	Provide the register name and registration number.	No

From: Page MJ, McKenzie JE, Bossuyt PM, Boutron I, Hoffmann TC, Mulrow CD, et al. The PRISMA 2020 statement: an updated guideline for reporting systematic reviews. BMJ 2021;372:n71. doi: 10.1136/bmj.n71

For more information, visit: http://www.prisma-statement.org/

Supplementary B: PICOS criteria for inclusion

	Criteria
P - patient	adult human patients with oropharyngeal carcinoma
I - intervention	all types of proton therapy
C - comparison	Comparison to baseline status or photon therapy
O - outcome	Patient-reported or quality of life measures
S – type of study	Randomised-controlled trials, cohort (prospective or retrospective). Exclude studies with no statistical comparisons (case study or case series) or reviews.

Supplementary C**: Search strategy using Pubmed and Scopus databases**

Database	Search string	Articles found
**Stage 1**		
Pubmed	("proton therapy"[MeSH Terms] OR ("proton"[All Fields] AND "therapy"[All Fields]) OR "proton therapy"[All Fields]) AND ("oropharyngeal neoplasms"[MeSH Terms] OR ("oropharyngeal"[All Fields] AND "neoplasms"[All Fields]) OR "oropharyngeal neoplasms"[All Fields] OR ("oropharyngeal"[All Fields] AND "carcinoma"[All Fields]) OR "oropharyngeal carcinoma"[All Fields])	107
Scopus	TITLE-ABS-KEY (oropharyngeal OR oropharynx) AND TITLE-ABS-KEY (proton AND therapy) AND TITLE-ABS-KEY (cancer OR carcinoma)	225
	Unique titles	252
**Stage 2**		
	Unique citations from selected articles not found in Stage 1	240
	Included	0
	Included from citations	1

Supplementary material D: Quality check of selected studies

	**Study**											
	Blanchard 2016			Sharma 2018			Manzar 2020			Sio 2016		
**Criteria**	**Yes**	**No**	Other (CD, NR, NA)*	**Yes**	**No**	Other (CD, NR, NA)*	**Yes**	**No**	Other (CD, NR, NA)*	**Yes**	**No**	Other (CD, NR, NA)*
1. Was the research question or objective in this paper clearly stated?	/			/			/			/		
2. Was the study population clearly specified and defined?	/			/			/			/		
3. Was the participation rate of eligible persons at least 50%?			CD			CD			CD			CD
4. Were all the subjects selected or recruited from the same or similar populations (including the same time period)? Were inclusion and exclusion criteria for being in the study prespecified and applied uniformly to all participants?		/			/			/			/	
5. Was a sample size justification, power description, or variance and effect estimates provided?	/				/			/				/

6. For the analyses in this paper, were the exposure(s) of interest measured prior to the outcome(s) being measured?	/	/	/	/
7. Was the timeframe sufficient so that one could reasonably expect to see an association between exposure and outcome if it existed?	/	/	/	/
8. For exposures that can vary in amount or level, did the study examine different levels of the exposure as related to the outcome (e.g., categories of exposure, or exposure measured as continuous variable)?	/	/	/	/
9. Were the exposure measures (independent variables) clearly defined, valid, reliable, and implemented consistently across all study participants?	/	/	/	/
10. Was the exposure(s) assessed more than once over time?	NR	NR	NR	NR
11. Were the outcome measures (dependent variables) clearly defined, valid, reliable, and implemented consistently across all study participants?	/	/	/	/
12. Were the outcome assessors blinded to the exposure status of participants?	/	/	/	/
13. Was loss to follow-up after baseline 20% or less?	CD	CD	CD	CD

14. Were key potential confounding variables measured and adjusted statistically for their impact on the relationship between exposure(s) and outcome(s)?	/	/	/	/

	**Study**					
	**Bagley 2020**			**Grant 2020**		
**Criteria**	**Yes**	**No**	Other (CD, NR, NA)*	**Yes**	**No**	Other (CD, NR, NA)*
1. Was the research question or objective in this paper clearly stated?	/			/		
2. Was the study population clearly specified and defined?	/			/		
3. Was the participation rate of eligible persons at least 50%?			CD			CD
4. Were all the subjects selected or recruited from the same or similar populations (including the same time period)? Were inclusion and exclusion criteria for being		/			/	

in the study prespecified and applied uniformly to all participants?						
5. Was a sample size justification, power description, or variance and effect estimates provided?		/			/	
6. For the analyses in this paper, were the exposure(s) of interest measured prior to the outcome(s) being measured?	/			/		
7. Was the timeframe sufficient so that one could reasonably expect to see an association between exposure and outcome if it existed?	/			/		
8. For exposures that can vary in amount or level, did the study examine different levels of the exposure as related to the outcome (e.g., categories of exposure, or exposure measured as continuous variable)?	/			/		
9. Were the exposure measures (independent variables) clearly defined, valid, reliable, and implemented consistently across all study participants?	/			/		
10. Was the exposure(s) assessed more than once over time?			NR			NR
11. Were the outcome measures (dependent variables) clearly defined, valid, reliable, and implemented consistently across all study participants?						
12. Were the outcome assessors blinded to the exposure status of participants?	/			/		

13. Was loss to follow-up after baseline 20% or less?		CD		CD
14. Were key potential confounding variables measured and adjusted statistically for their impact on the relationship between exposure(s) and outcome(s)?	/		/	

*CD, cannot determine; NA, not applicable; NR, not reported
